# Addressing Antimicrobial Resistance: An Overview of Priority Actions to Prevent Suboptimal Antimicrobial Use in Food-Animal Production

**DOI:** 10.3389/fmicb.2016.02114

**Published:** 2017-01-06

**Authors:** Guillaume Lhermie, Yrjö T. Gröhn, Didier Raboisson

**Affiliations:** ^1^Department of Population Medicine and Diagnostic Sciences, College of Veterinary Medicine, Cornell University, IthacaNY, USA; ^2^BioEpar, Oniris, Institut National de la Recherche Agronomique (INRA)Nantes, France; ^3^Interactions Hôtes Agents Pathogènes, Institut National de la Recherche Agronomique (INRA) – Ecole Nationale Vétérinaire Toulouse, Université de ToulouseToulouse, France

**Keywords:** antimicrobials, antimicrobial resistance, veterinary precision medicine, public policies, livestock production, agricultural economics

## Abstract

The growing concern regarding emergence of bacteria resistant to antimicrobials and their potential for transmission to humans via animal production has led various authorities worldwide to implement measures to decrease antimicrobial use (AMU) in livestock production. These measures are influenced by those implemented in human medicine, and emphasize the importance of antimicrobial stewardship, surveillance, infection prevention and control and research. In food producing animals, unlike human medicine, antimicrobials are used to control diseases which cause economic losses. This major difference may explain the failure of the public policies implemented to control antimicrobial usage. Here we first review the specific factors influencing AMU across the farm animal sector and highlighting the farmers’ decision-making process of AMU. We then discuss the efficiency of existing regulations implemented by policy makers, and assess the need for alternative strategies, such as substitution between antimicrobials and other measures for infectious disease control. We also discuss the interests of regulating antimicrobial prices. Finally, we emphasize the value of optimizing antimicrobial regimens, and developing veterinary precision medicine to achieve clinical efficacy in animals while limiting negative impacts on public health. The fight against antimicrobial resistance requires both a reduction and an optimization of antimicrobial consumption. The set of actions currently implemented by policy makers does not adequately address the economic interests of farmers’ use of antimicrobials.

## Introduction

Since the discovery of sulfonamides in 1932, antimicrobials have been used across the human and animal health sectors to cure infectious diseases. The global rising demand for animal protein for human feeding will potentially lead to a massive increase in **AMU** in food-animal production, estimated to rise by 67% between 2010 and 2030. However, because of differences between national regulatory frameworks surrounding antimicrobial sales, there are only limited data regarding AMU, particularly in low- and middle- incomes countries (Van Boeckel et al., 2015). In 2012, 26 European Union countries’ average consumption of antimicrobials was respectively 116.4 and 144.0 milligrams per kilogram of estimated biomass in humans and animals (European Centre for Disease Prevention, and Control [ECDC]/European Food Safety Authority [EFSA]/European Medicines Agenc [EMA], 2015). As in human medicine, AMU in veterinary medicine, even when rational, might lead to the selection of genetic material encoding for bacterial resistance. The selection of resistant bacteria, zoonotic pathogens or commensals represents a particular public health hazard as it can be transferred to humans via direct contact or indirectly via the food chain (Aarestrup and Wegener, 1999; Singer et al., 2003).

The quantitative impact of AMU in veterinary medicine on public health remains difficult to assess (Singer and Williams-Nguyen, 2014). However, based on its potential impact, most countries from Northern Europe implemented specific plans to decrease AMU in veterinary medicine. The practical measures adopted are very similar to those observed in human medicine. They rely on general concepts on the control of AMU; four topics are systematically involved, among others: (i) stewardship, (ii) surveillance, (iii) infection prevention and control and (iv) research. The interests of such measures have been widely documented in human health (Edwards and Gould, 2012; Premanandh et al., 2016). In accordance with the One Health concept, the importance of controlling AMU in the animal sector has also been highlighted (Dar et al., 2016; Premanandh et al., 2016). Prescription of antimicrobials in companion animals basically follows an individual diagnosis of illness. In food animal production, antimicrobials are used in three different situations: (i) curative treatment of diseased animals, (ii) metaphylactic treatment of a group of animals, meaning treatment of diseased and healthy animals belonging to the same group in case of high risk of contagious disease, and (iii) prophylactic treatment of a group of healthy animals, considered at high risk of developing the disease in the case of no prophylaxy. Since the quantities of antimicrobials used in companion animal medicine are very low compared to those used in the overall animal sector, farm animals are recognized as the main source regarding AMR. As such, companion animals will not be further considered in the present study.

Since the ban of antimicrobials as growth promoters in the European Union in 2003, followed by the USA in 2015, various sets of measures regarding AMU have been implemented in veterinary medicine, generally mixing regulations and voluntary approaches (European Union, 2003; Food and Drug Administration, 2015). These measures have led to spectacular decreases in antimicrobial consumption. For example, a 58.1% decrease in antimicrobial consumption in food producing animals was observed between 2009 and 2014 in the Netherlands (MARAN, 2015). In France, the global exposure to antimicrobials in veterinary medicine decreased by 15.7% between 2007 and 2013 (Chevance and Moulin, 2014).

Yet, these results hide large differences among species, administration route and classes. For instance, in the Netherlands in 2014, the veterinary sales of penicillins, third and fourth generation cephalosporins, fluoroquinolones, amphenicols and macrolides and lincosamides increased by 10, 4.3, 2.4, 18, and 12%, respectively, in spite of a massive decrease of these classes in the previous years; in the meantime, sales of tetracyclines, trimethoprim/sulphonamides, pleuromutilins, polymyxins and aminoglycosides dropped (MARAN, 2015). In France, between 2007 and 2013, veterinary prescription of antimicrobials administered per oral route decreased by 24% whereas prescription of parenteral antimicrobials increased by 9%. The exposure decreased by 28, 10, 13% in pigs, companion animals and poultry, respectively, but slightly increased (+0.2%) in cattle (Chevance and Moulin, 2014). Altogether, these observations show that the decrease in AMU is multifactorial and follows complex and various steps and ways. Therefore, the risk that the above mentioned policies fail to tackle AMR is high, as has been suggested in human health (Wallinga et al., 2015). Furthermore, as several measures are implemented simultaneously, the evaluation of their individual impact remains challenging.

The aims of this study are to provide an overview of identified factors influencing AMU across the farm animal sector, to discuss the effectiveness of existing measures minimizing AMR, to evaluate the possibilities of substitution between antimicrobials and other production factors, and to discuss the need to develop research programs to optimize antimicrobial efficiency in a One Health perspective.

## Factors Influencing Antimicrobial Use: the Crucial Issue of Farmer’S Decision Making

### Occurrence of Infectious Diseases and Pathogen Expression at the Farm Level

**FIGURE 1:**
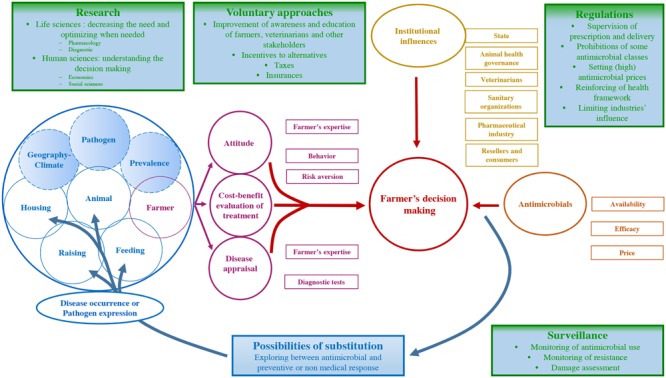
**Factors influencing antimicrobial use in food-animal production, and priority actions preventing their suboptimal use**.

Most infectious diseases occur in cases of simultaneous presence of an infectious agent and a susceptible host, the latter being influenced by exogenous and endogenous risk factors (**Figure 1**, blue circles). Exogenous risk factors are imposed on farmers; they have little, if any, control over them. Pathogens and their regional prevalence, climate or general factors such as changes in markets represent exogenous factors of infectious diseases. For example, respiratory infections affecting young cattle are more frequent in winter (Cernicchiaro et al., 2012). These factors directly contribute to the basal level of infectious diseases observed in a given production system (Raboisson et al., 2012). Similarly, a basal level of antimicrobials is used in each given production system, almost proportionally to the basal level of infectious disease.

The basal level of infectious disease (and its associated AMU) is the direct result of the expression of exogenous risk factors. This level can be defined considering a good (perfect) management of the farmer facing these –changing– exogenous factors. Because the production systems are complex and farmer decisions are not always optimal (see below), a suprabasal level of infectious disease and AMU is often considered as being acceptable by professionals. This level represents the health convention (or health standards) of the given livestock system (Raboisson, 2011), meaning an accepted or approved target against which others are judged or measured. The health convention for a given livestock system considers at least a health standard for diseases occurrence and a health standard for AMU. Many farms do not reach these health standards, mainly due to endogenous factors.

The endogenous risk factors of infectious disease relate directly to the decisions of farmers who can control them. They include how the farmer adapts to the exogenous risk factors of a given system as well as how he reacts to the occurrence of disease. These two topics represent the preventive and curative aspects of the disease management. Both aspects are the quintessence of a farmer’s role in AMU. For instance, animal nutritional status, housing and raising conditions, feed quality and quantity, and choice of breed are examples of endogenous factors. Poor decision making in regards to endogenous risk factors such as the absence of quarantine, having a shared air space for several groups of calves, and the lack of clinical examination upon arrival at the farm may lead to development of respiratory diseases which have also been associated with increased incidence of antimicrobial treatments (Lava et al., 2016).

Interestingly, most endogenous risk factors are, and behave as, production factors. This means that variations in these endogenous and production factors affect both the quantity of produced foodstuffs (outputs) such as milk or meat and the risk of disease occurrence. For example, implementing quarantine requires extra pens that will (hopefully) only be used a small part of the time. Their presence decreases the risk of disease in the herd. In the absence of disease, these extra pens are unnecessary production factors, which decrease the overall benefit to the farmer.

At the farm level, the farmer holds a key position, with a dual role of decision maker and risk factor *per se*. The farmer’s choices in the herd management will therefore directly and indirectly influence the disease occurrence and consequently AMU.

### On Farm Factors Influencing the Farmer’s Attitude toward Disease

The occurrence but also the threat of infectious disease (see below) represents the first step conditioning the implementation of an antimicrobial treatment. Considering this occurrence, the farmer establishes a response influenced by farm factors (**Figure 1**, purple boxes and circle), under a given institutional context (**Figure 1**, golden boxes). The farmer’s decision making is primarily influenced by three components: (i) the cost-benefit analysis of the treatment for the disease, (ii) the farmer’s expertise, attitude toward risk, and behavior, and (iii) his/her ability to detect the disease.

#### Cost-Benefit Analysis

Antimicrobials are used to cure infectious diseases in both human and veterinary medicine. In addition to the shared therapeutic objective, AMU also achieves in veterinary medicine an economic objective through the optimization of the farm benefits. Depending on the characteristics of the diseases (infectiousness, morbidity, lethality) and their consequences (individual, local, global level), the losses associated vary massively. As an example of highly and fast contagious disease, Pendell et al. (2015) assessed the costs of a hypothetic outbreak of Foot and Mouth Disease in the United States, ranging from $16 to $140 billion. On the other hand, the costs of clinical mastitis generally affect only the aﬄicted herd, representing roughly a cost of $224 to $275 per case (Steeneveld et al., 2011). Many assessments of losses or total cost of diseases exist but few clearly evaluate the profitability of different strategies of management including use of antimicrobials as curative or preventive treatment. Nevertheless, in spite of formal demonstration, the comparison between the price of antimicrobials and the cost of diseases suggest a highly profitable use of antimicrobials for many of the diseases (but probably not all), at least in first intention therapy.

Food animal production is an economic process where the farmer’s first goal is animal foodstuffs trading. In this production process, disease occurrence appears as damage, and recourse to antimicrobials tends to limit the losses generated by the disease. Therefore, the farmer may consider antimicrobials as a production factor, and the decision to use antimicrobials may be based on the expected financial return associated with the treatment. The profitability associated with the use of antimicrobials is based on the cost of the treatment and on the expected return associated with damage control (Tisdell, 1995). The control of the damage becomes an economic process, in which the antimicrobial treatments generate an additional output at least equivalent to saved losses (compared to no treatment). The economic rationality should lead the farmer to use antimicrobials only when profitable. In cases of diseases already present, the choice is made regarding the expected saved losses (depending on expected antimicrobial efficacy and on the extended impact of the disease, i.e., severity, risk of spread of disease or mortality without treatment) and the price of the treatment. In addition to these items, the implementation of a preventive treatment is also based on the probability of occurrence of the disease in the future. This means that the price of antimicrobials represents a key component of AMU, for both prevention and curative treatments. In many countries, the high efficacy of antimicrobials and their relatively low cost make their use favorable. To sum up, for a given livestock system and a given disease, an economic optimum of disease control is defined as a function of the level of threat of the disease in absence of treatment, the risk of being infected/ill, the expected efficacy of the therapy and the cost of veterinary service. The value of the production (outputs) is also a factor influencing AMU since the higher the price of the output, the higher the economic impact of the disease in the case of no treatment. AMU is sensitive to the antimicrobial price. A decrease in the price of antimicrobials, which occurred with the introduction of fluoroquinolone generics on the French market, was for instance associated with a significant increase in the amount of fluoroquinolones consumed in poultry production (Chevance and Moulin, 2009).

#### Farmer’s Expertise, Attitude toward Risk, and Behaviors

During the decision-making process, the farmer balances the cost of antimicrobials with the expected benefits of the treatment. This refers to the “perceived benefits” developed in the 1950’s in the Health Belief Model to understand behaviors of patients’ response to a disease (Rosenstock, 1974). When facing a threat, the farmer decides to use one antimicrobial according to his/her perception of efficacy, based on regulatory guarantees such as market authorization, but also based on his/her own beliefs (Grein, 2012). Farmers’ choices were shown to be strongly influenced by personal beliefs and perceptions (Hadar and Fischer, 2008; Kristensen and Jakobsen, 2011). When facing a given disease threat, the farmer subjectively evaluates the potential impact of the disease in cases of no treatment and the net return. Before any administration of preventive treatment (with antimicrobials or not) in the presence of risk factors of disease, the farmer subjectively evaluates (i) the real risk of disease occurrence, (ii) the potential impact of the disease in case it occurs and (iii) the technical possibilities of curative treatment if disease occurs. All these evaluations vary according to his/her technical expertise (knowledge and skills), his/her attitude toward risk and his/her behaviors.

Technical expertise was reported to be associated with age, education level and level of antimicrobial consumption. Danish pig farmers with less than 4 years of practical experience detected clinical diarrhea more accurately than farmers with more than 4 years of practical experience (Pedersen and Strunz, 2013). Similarly, young Belgian farmers were more likely to implement biosecurity measures in their herd, leading to a decrease in antimicrobial consumption (Laanen et al., 2013). The farmers’ age was likely to be a proxy for recent education. Low education and poor knowledge of farmers regarding AMU were associated with increased AMR and zoonotic infections in a Sudan study (Eltayb et al., 2012). Both herd characteristics and farm management impact the level of AMU, and explain heterogeneity within farms: for instance, 25% of the French poultry herds consumed between one and two thirds of the total antimicrobial amount (Chauvin et al., 2012).

A farmer’s risk aversion also leads to higher AMU, when the farmer expects a decreased risk in the production system as he uses antimicrobials. Risk aversion is particularly observed during peculiar stress or economic crisis. The farmer seeks to secure his/her income while avoiding a major epidemic, even if his/her income (decreased by additional antimicrobial costs) remains lower than the optimal one. Such behaviors have been described in a survey conducted in rabbit production, showing a negative correlation between income level and AMU (Chauvin et al., 2011). The influence of attitude toward risk was also evidenced in beef cattle farmers facing climatic hazard or decrease in meat prices (Mosnier, 2009). Since respiratory diseases are the key disorder in this line of production, beef farmers with a strong risk aversion are more likely using antimicrobials –including for prevention– limiting the probability of a massive respiratory outbreak.

Farmer’s behaviors are also influenced by non-economic factors, such as values and motivations. The farmer’s decisions to follow (or not) the recommendations can partially be explained through social sciences and the different categories of farmers (Garforth, 2015). A recent study conducted on sheep farmers’ response to foot rot focused on the effects of personality, emotions such as empathy and general attitude on the treatment strategy. The authors found that sheep had an increased risk of lameness when their farmers demonstrated negative emotions and hopelessness toward foot rot, and that farmers’ personality and emotions are associated with their differences in management of foot rot (O’Kane et al., 2016).

#### Disease Appraisal

Disease diagnosis relies on a combination of farmers’ knowledge and available diagnostic tools. A major difference from human medicine is that antimicrobial therapy is often implemented empirically, based upon the concurrent presence of epidemiological and clinical factors, and rarely involves laboratory testing confirming the presence of bacterial infection. Availability and cost of tests at the animals’ side seem to limit their use, particularly when the results of these tests cannot be extrapolated to the entire herd and may also need to be frequently repeated (Speksnijder et al., 2015a). The moderate costs of treatment – including antimicrobials – also limit the use of tests before treatment. For individual animal treatment (internal medicine), additional costs of laboratory testing substantially increase the total costs of disease treatment supported by the farmers, and therefore probabilistic therapeutic approaches are common. In population medicine, the use of tests is more frequent since their marginal costs are decreased.

Absence of specific and sensitive tests is not rare for food producing animal diseases. As an example, absence of a specific field diagnostic test for bovine respiratory disease has been reported as a limitation in diagnosing cattle and might lead to an overconsumption of antimicrobials to prevent the appearance of infection (Taylor et al., 2010).

### Institutional Influences

Decision making also depends on institutional influences, such as technical environment, regulations and food chain organization (**Figure 1**, golden boxes).

In several European countries, voluntary and regulatory actions were implemented by policy makers, involving farmers’ health management associations and the veterinary profession. Several European countries decided on a ban of the preventive use of CIAs. Complementary mandatory measures were implemented, including the use of susceptibility testing before any prescription of CIA for curative purposes. To avoid competition among veterinary practices and therefore potential pressure in antimicrobial prescription and to promote farmer-veterinary relationships and farmer’s education, Dutch farmers were asked by the government to choose only one veterinary practice to provide oversight and prescription in their herds (Speksnijder et al., 2015b). In France, a “Plan Ecoantibio” program was initiated in 2012 and lasts until 2017. This is a voluntary approach program fixing an objective of a 25% decrease in AMU. It is coordinated by the government and emphasizes farmers’ stewardship (Lhermie et al., 2015). In France, the United Kingdom, the Netherlands and Belgium, treatment guidelines were developed, supporting veterinarians in their therapeutic decision-making.

The decision making of the farmer also depends on access to antimicrobials (**Figure 1**, orange boxes). In most high-income countries, veterinary antimicrobial delivery is submitted to a regulated system, involving prescribers (veterinarians) and suppliers (veterinarians, pharmacists, others). The regulations surrounding antimicrobial prescription and delivery should theoretically ensure their strict usage and limit unjustified consumption from a medical point of view. The question of the influence of farmers on veterinarian’s prescription is poorly known, but certainly not dissimilar from the influence of patients on their physicians, which has already been reported (Avorn and Solomon, 2000; Orzech and Nichter, 2008).

For certain antimicrobial classes such as CIA, policies were implemented to ban or limit their use in animal health (Collignon et al., 2009). However, in many low-income countries, weakly or poorly regulated practices surrounding manufacturing and access to drugs might encourage inappropriate use and therefore reduce stewardship (Dar et al., 2016). In countries with weak regulations, it has been shown that a major part of the sales were performed over the counter, resulting in potential incorrect dosages and non-observance of withdrawal periods (Shryock, 2012). Lastly, they increase the possibilities of counterfeit drugs, without guarantee of efficacy.

People outside the farm and more broadly the farm network (so-called institutional context of the farm) may also influence AMU. Discrepancies in AMU were observed in cattle feedlots, depending on clinical circumstances and on actors in the feedlot network (McIntosh and Dean, 2015). The influence of the social network and citizenship behavior has also been reported to influence decisions in a survey focused on farmers’ attitudes toward BVD control in the UK (Heffernan et al., 2016).

Consumers have already started to indirectly influence food animal production systems and their associated AMU. In high-income countries, the growing demand of foodstuffs produced respecting environmental, animal welfare and public health issues has become a strong driver of AMU decrease. Consumers’ impact is likely to grow, including strict antimicrobial restrictions in animal production. For instance, McDonalds’ USA recently announced that the goal set in 2015 to “only serve chicken not treated with antibiotics important to human medicine” was achieved in July 2016 (McDonalds’, 2016).

The question of the potential conflict of interests for veterinarians as both prescribers and suppliers has been recently addressed (Rosbach, 2012). France and the Netherlands decided to maintain this dual status for veterinarians (Dahan, 2013). The Danish government implemented advisory roles to compensate veterinarians for income loss due to removing their status as suppliers (Dar et al., 2016). To our knowledge, no study has assessed the influence of the veterinary pharmaceutical industry on veterinarians’ and farmers’ attitudes. In human medicine, interactions between industry and physicians have already been described Spurling et al. (2010), and Austad et al. (2011).

## Priority Actions to Minimize the Impact of Veterinary Antimicrobial Use on Public Health

Tackling AMR relies on a set of measures that have been widely reviewed Jarlier et al. (2012). Priority actions include strengthening industrial and academic research, regulation of the antimicrobial market, surveillance of the use, and improvement of awareness and education of healthworkers, farm personnel and the public. However, the specificity of use of antimicrobials as production factors in food animal production suggests that substitution between antimicrobials and other production factors have to be considered. Green and blue rectangles in the **Figure 1** represent the different priority actions.

### Substitution between Antimicrobials and Non-medical or Preventive Tools

Substitutions between antimicrobials and other items are likely to represent the most important leverage of decrease in AMU in veterinary medicine. The substitution can take place for curative or preventive treatments and should be seen as short or long term, meaning for the current or future production cycles. Substitutions are based on the endogenous risk factors of infectious disease and of AMU, previously defined as production factors taking place in an economic process. In other words, the economic substitution between AMU and another practice could be economically perfect and even be financially in favor of the non-antimicrobial practice.

In the case of declared disease, antimicrobials remain an efficient damage control tool, with few possibilities of substitution. Therefore, the overall antimicrobial demand is highly rigid. The antimicrobial consumption may be limited by early diagnosis of disease to limit the contagion to the whole herd or group. The demand for a given antimicrobial is not totally rigid since a substitution exists between different classes of antimicrobials. For many diseases, the cost-benefit ratios –based on low cost, easy-to-use and high efficacy– are in favor of the new generations of antimicrobials even in first intent, although older classes may be “sufficient.”

The difficulties in estimating the economic value of livestock and the volatility of output prices and production factors limit non-medical alternatives, even if these appear societally more relevant, given the public health negative externality associated with AMU.

For each situation with a possible substitution, the question of its economic interest remains to be asked. The substitutability among production factors encourages the use of inputs with fast return on investments such as antimicrobials, whose effectiveness is guaranteed under standardized conditions and which secures farmers’ income. In addition, antimicrobials remain an easy-to-use short-term damage control tool, whereas many other factors exhibit their efficiency over a longer time frame. This is all the more true for last generations of antimicrobials.

In the case of a preventive approach, the substitution between preventive antimicrobial and preventive non-antimicrobial (vaccine) or other non-health factors of production (food, housing) can be easily considered. The application of good rearing practices and higher biosecurity, i.e., changing clothes and shoes before entering a facility, was found to be associated with lower antimicrobial consumption in pigs (Chauvin et al., 2005a; Laanen et al., 2013). Similarly, vaccination of calves at arrival –or better yet, before arrival– reduces the number of curative treatments of respiratory diseases of young cattle (Assie et al., 2009).

Finally, the substitution between antimicrobials and other production factors requires anticipation of risk of the health event, based on disease prevalence and presence of endogenous and exogenous factors. The farmer has to pick one out of two strategies considering equilibrium between the costs of treatments and the costs of losses. In the preventive approach, the costs of treatments are known (the number of animals to treat and the costs of drugs). In the curative approach, there remains uncertainty regarding both costs of treatments and costs of losses; if the disease does not occur, the farmer maximizes his/her profit since no animals require a treatment, but in the case of disease occurrence, it is likely that global costs of disease are higher than costs of a preventive approach. Depending on the epidemiologic form of the disease (epidemic or endemic), and therefore its consequences in the future, the strategies can vary: short term, high losses associated with a disease outbreak should lead the farmer to favor a preventive approach, whereas long term or losses spread out over time associated with endemic diseases could lead to a passive strategy.

At the farm level, budgetary constraints lead to allocations of monetary resources to highly profitable strategies of disease control. These constraints limit farmers’ investments (even more profitable in the long term) and increase a little more their antimicrobial demand. Lastly, even if antimicrobials do not provide any return on investment in the absence of future disease, their technical effectiveness easily secures the production process productivity. Hence, they remain powerful damage control tools, highly resistant to budget constraints.

To address this issue, future research is needed to assess the impact of alternative strategies using fewer antimicrobials. Economic evaluation of infectious disease management reducing AMU, at both herd and societal levels, should be performed over time, to consider mid-range and long term benefits. Given the interdependence among production factors and among farmers, health workers, retailers and regulators, approaches merging biology, social sciences, and modeling allow consideration of the complexity of the whole system (Grohn, 2015).

### Awareness and Education

Regarding the importance of a farmer’s knowledge in the decision making process, one can assume that all the measures implemented to raise awareness and responsible AMU should be encouraged, although little evidence of the benefit of such measures in veterinary medicine exists.

Improvement of education and awareness can be attained by proximity technical transfer or as part of coordinated actions. These two methods are based on the constant interactions between farmers and organizations – public or private – playing a role in health care (Chilonda and Van Huylenbroeck, 2001; Raboisson et al., 2012). The major influence of veterinarians on farmers’ AMU has been reported in several papers (Kaneene and Miller, 1992; Chauvin et al., 2005b; McIntosh and Dean, 2015; Speksnijder et al., 2015a). In some cases, institutions can coordinate collective action to face a collective problem (Ostrom, 1990). In human medicine, for example, consumer awareness through communication campaigns coordinated by the French government (campaign “Antibiotics are not automatic”) led to a decrease in volumes of antibiotics consumed (Sabuncu et al., 2009). Educating practitioners in small groups led by an expert also results in a significant reduction of drug consumption (Madridejos-Mora et al., 2004; Ranji et al., 2008). Conversely, wide diffusion of treatment guidelines appears to produce less tangible results (Faryna et al., 1987; Al-Momany et al., 2009).

The success of campaigns seems to rely on their adaptation to local conditions, the existence of a network, and support from governmental and professional institutions (Huttner et al., 2014). In Mexico, a bottom–up approach developing a national strategy to fight AMR partially failed, because of a lack of regulatory support (Zaidi et al., 2015). Hence, such strategies might not be adequate in low-income countries (Diazgranados et al., 2008).

In the animal health sector, research documenting the impacts of the implemented measures is lacking and should be encouraged, in addition to the national monitoring programs of antimicrobial consumption which depict the global trends in antimicrobial consumption. Furthermore, recent research suggests that a deep understanding of the decision making process is needed, as it can explain success or failure of policy interventions (Rat-Aspert and Fourichon, 2010; Garforth, 2015; O’Kane et al., 2016).

### Regulations

Across countries, organization of the veterinary drug delivery system varies. Therefore, governments have implemented specific measures in accordance with their local scheme. Denmark, the Netherlands and France have implemented restrictive measures on usage of CIA, leading to a massive decrease in consumption of these targeted antimicrobial classes (Aarestrup, 2012; Grohn, 2015; Lhermie et al., 2015; Speksnijder et al., 2015a). However, the impact of such restrictions on misuse or overconsumption is questionable. Indeed, for a given condition, it is likely that a CIA will be substituted by a non-CIA, which ultimately (i) will not decrease exposure of animals to antimicrobials, and (ii) will have only little effect on the externality [AMR of commensal bacteria, due to the existence of cross-resistance between the different classes (Carattoli, 2009)].

Regulation of antimicrobial sales prices has been surprisingly understudied. To our knowledge, sales prices are generally not regulated. Given the principle of cost/benefit to the farmer and the close relationship between antimicrobial cost and usage, discussed earlier, setting high prices to antimicrobials may certainly help limit overconsumption.

### Financial Alternatives to Prices’ Control: Taxes and Insurances

A first alternative to prices’ control consists of setting a tax on antimicrobial sales, on the basis of costs developments supported by pharmaceutical industries (Vagsholm and Hojgard, 2010). A Pigovian tax system could also be implemented (Pigou, 1920). Its implementation nevertheless requires a quantitative evaluation of the costs of AMR associated with AMU. The complex evaluation of these costs might be a factor explaining that the countries introducing taxes in their set of measures chose to tax consumers. In Denmark, a range of differentiated taxes on antimicrobials have been applied since 2013 in order to promote the use of vaccines instead of antimicrobials with a specific focus on CIA. The tax rates vary between drugs: 0% for vaccines, 0.8% for narrow-spectrum penicillins and other veterinary medicines, 5.5% for other veterinary antimicrobials and 10.8% for CIA. However, a recent report mentioned that even if the taxes generated fund financing activities on AMR, they did not greatly affect antimicrobial consumption (European Commission, 2016).

Since June 2014, Belgium has implemented taxes on all antimicrobials sold for use in veterinary medicine. The amount is calculated as a function of the total quantity of active ingredient (1.75/kg), with an increase of 1.5 fold for CIA (European Commission, 2015).

Increasing antimicrobials prices with taxes may provide interesting changes in the case of preventive AMU, based on substitution principles. Yet, such alternatives in the case of curative AMU may have limited effects. Given the need for antimicrobials to limit economic losses, it is likely that in the context of curative individual usage of antimicrobials, a higher cost does not lead to a significant decrease in AMU. In this context, demand for antimicrobial remains rigid at their current average prices.

Another alternative consists of the development of insurance policies. Infectious diseases generate production risks, which can be covered by insurance fewer than two conditions: independence of risks and symmetry of information. In animal production, the literature remains sparse compared to that of crop production. Among the several insurance options, income insurance is reported to be potentially more attractive to farmers than other forms of insurance (e.g., yield, price), because it deals with losses affecting the farmer’s welfare more directly (Meuwissen et al., 1999). The insurance premium could rely on net farm revenues. However, insurance of individual income risks raises problems of moral hazard and adverse selection. As described above, potential losses associated with infectious diseases are explained by exogenous but also endogenous risk factors, the latter being influenced by the choices of the farmer, which can be different in the case of insurance subscription. Furthermore, income can be easily manipulated in the farmer’s balance sheet. These two factors make the calculation of the probability distribution of a threat and therefore the establishment of the insurance premium complex.

### Surveillance of AMU and AMR

Surveillance represents a key component of the control of AMR (Premanandh et al., 2016). The World Health Organization developed standards to detect emergence of resistance. In parallel, several countries implemented monitoring of resistance among commensal and zoonotic bacteria. These measures should be associated with the monitoring of antimicrobial consumption. Harmonization of the methods of collecting data is in progress in Europe, as comparison between countries could be a major driver for change (Dar et al., 2016). In low-income countries, monitoring of antimicrobial consumption and resistance is rare and international organizations have a major role to play to address these issues (Aidara-Kane, 2012).

### Research and Innovation

#### Optimizing Actual Treatments

It is very likely that new antimicrobial drugs will be restricted to human medicine (Jorgensen et al., 2016). Hence research in veterinary antimicrobial therapy should be performed to optimize dosage regimen of existing drugs, as functions of the targeted bacteria, host species, and according to PK/PD considerations. Re-evaluation of antimicrobial dosage regimens, considering updated PK/PD requirements, constitutes a first step to limit the impact of antimicrobials on commensal floras. For example, recently in veterinary medicine, the marbofloxacin dosage regimen was re-evaluated for treatment of Gram negative infections and the current labeled dose of 10 mg/kg given in a single injection was added to the first label approved dosage of 2 mg/kg over 3–5 days, leading to a decrease in the time of residence of the drug in the body while maintaining clinical efficacy (Valle et al., 2012). Dosage regimens of existing drugs previously determined according to results of clinical trials can also be evaluated based on standard PK/PD concepts, as performed with tulathromycin in calves (Toutain et al., 2016b).

Innovations in veterinary AMU should also aim to limit the impact of antimicrobials on the commensal flora (especially the digestive flora) of the treated animals, without reducing their curative efficacy on the targeted bacterial infection. A recent review depicted the PK/PD characteristics of the ideal antimicrobial for food-producing animals (Toutain et al., 2016a). This antimicrobial should be administered orally, and antimicrobials with a high bioavailability, with limited interactions with compounds of the digestive tract, should be preferred. In the case of parenteral administration, long-acting compounds with a low clearance, ensuring long half-life elimination, or long-acting formulations limiting the impact on the digestive flora, are recommended.

#### Veterinary Precision Medicine

In human medicine, Personalized Medicine is defined as a form of medicine built on information about patients’ genes and their environment, aiming to refine diagnosis, to select the most appropriate treatment and increase the patient’s chances of recovery (Feero and Guttmacher, 2014; Peer, 2014). In parallel, Precision Agriculture emerged in the late 20th century, fulfilling an objective of optimization of agricultural practices while considering their environmental impacts (McBratney et al., 2005). In crop science, digital technologies (satellite mapping, climate and epidemic alarm systems) provide farmers tools for supporting decision making. These tools allow for example, limitation of consumption of fertilizers or pesticides. In animal production, precision farming is defined as “the use of technology for monitoring physiological indicators, behavioral or production on animals to improve herd management strategies and breeding performance” (Bewley J., 2010).

Veterinary precision medicine, which we can define as an optimized preventive or curative therapeutic approach (right animal, right drug, right dose, right time) based on identification of biomarkers of disease and use of technologies of disease monitoring, may provide strong leverage of AMU rationalization. It relies on availability of diagnostic tools allowing one to quickly identify the pathogen and the bacterial load.

In field conditions, the lack of cheap and practical diagnostic tools constitutes a major barrier to rationalization of antimicrobial treatment, and innovation in this domain should be firmly encouraged. Optimization of the antimicrobial regimen can also be achieved by combining early detection of disease with a low antibiotic usage regimen. New techniques of bovine respiratory diseases diagnostics, such as cough captors or ruminal temperature boluses, can be used to monitor early signs of illness before appearance of clinical signs (Timsit et al., 2011a,b). Recent studies conducted with mice and calves suggested that a decrease in the administered dose of fluoroquinolone allowed achievement of similar bacterial cure, when low bacterial inocula are targeted and the disease is detected early (Ferran et al., 2011; Lhermie et al., 2016). These protocols significantly decrease the amount of antibiotic required to reach a similar clinical efficacy.

## Conclusion and Prospects

The fight against AMR requires reduction and optimization of antimicrobial consumption. The set of measures currently implemented overlooks the economic interests of farmers regarding their use of antimicrobials. The objectives of usage decrease targeted by policy makers remain a signal of importance of the AMR issue, but do not influence individual choices. Priority actions encouraging sustainable AMU, considering both societal issues and farmers’ constraints, should be targeted. Exploring the interests of alternatives and possibilities of optimization of AMU should be encouraged. In parallel, the development of initial and postgraduate education programs of farmers and health workers regarding antimicrobial stewardship, as well as an evaluation of the benefits of such measures, should be conducted.

## Author Contributions

GL, YG, and DR: Contributed to the conception of the work; drafted the work or revised it critically for important intellectual content; approved the final version to be published; agree to be accountable for all aspects of the work in ensuring that questions related to the accuracy or integrity of any part of the work are appropriately investigated and resolved.

## Conflict of Interest Statement

The authors declare that the research was conducted in the absence of any commercial or financial relationships that could be construed as a potential conflict of interest.

## Abbreviations

AMR: antimicrobial resistance
AMU: antimicrobial use
CIA: critically important antimicrobials
PK/PD: pharmacokinetics/pharmacodynamics
